# Development and technical validation of an ultrasound nebulizer to deliver intraperitoneal pressurized aerosols in a rat colon cancer peritoneal metastases model

**DOI:** 10.1186/s12885-022-09668-0

**Published:** 2022-05-21

**Authors:** Jonathan R. Buggisch, Daniel Göhler, Julien Sobilo, Stéphanie Lerondel, Günther A. Rezniczek, Michael Stintz, Andreas Rudolph, Nicolas Tabchouri, Sébastien Roger, Mehdi Ouaissi, Urs Giger-Pabst

**Affiliations:** 1 University of Münster, Medizinische Fakultät, Münster, Germany; 2 CNRS UPS44, CIPA, PHENOMIN-TAAM, Orléans, France; 3 Research Group Mechanical Process Engineering, Institute of Process Engineering and Environmental Technology, Technische Universität Dresden, Dresden, Germany; 4 Technologie-orientierte Partikel-, Analysen- und Sensortechnik, Topas GmbH, Dresden, Germany; 5 Department of Obstetrics and Gynecology, Marien Hospital Herne, Ruhr-Universität Bochum, Bochum, Germany; 6 Department of Digestive, Oncological, Endocrine, Hepato-Biliary, Pancreatic and Liver Transplant Surgery, University Hospital of Tours, Lille, France; 7 EA4245 Transplantation, Immunologie, Inflammation, Université de Tours, France; 8 Institut Universitaire de France, Paris, France; 9 University of Applied Science Düsseldorf, 40489 Düsseldorf, Germany

**Keywords:** Peritoneal metastasis, Human colorectal cancer cells, Orthotopic rat model, Ultrasonic nebulizer, Intraperitoneal pressurized aerosol

## Abstract

**Background/aim:**

To develop and validate a nebulizer device for anti-cancer research on pressurized intraperitoneal aerosol supply in a preclinical peritoneal metastases (PM) rat model.

**Material and methods:**

For aerosol generation, an ultrasonic nebulizer (USN) was modified. Aerosol analyses were performed ex-vivo by laser diffraction spectrometry (LDS). Intraperitoneal (IP) ^99m^technetium sodium pertechnetate (^99m^Tc) aerosol distribution and deposition were quantified by in-vivo single photon emission computed tomography (SPECT/CT) and compared to liquid IP instillation of equivalent volume/doses of ^99m^Tc with and without capnoperitoneum. PM was induced by IP injection of HCT116-Luc2 human colon cancer cells in immunosuppressed RNU rats. Tumor growth was monitored by bioluminescence imaging (BLI), ^18^F-FDG positron emission tomography (PET) and tissues examination at necropsy.

**Results:**

The USN was able to establish a stable and reproducible capnoperitoneum at a pressure of 8 to 10 mmHg. LDS showed that the USN provides a polydisperse and monomodal aerosol with a volume-weighted diameter of 2.6 μm. At a CO_2_ flow rate of 2 L/min with an IP residence time of 3.9 s, the highest drug deposition efficiency was found to be 15 wt.-%. In comparison to liquid instillation, nebulization showed the most homogeneous IP spatial drug deposition. Compared to BLI, ^18^F-FDG-PET was more sensitive to detect smaller PM nodules measuring only 1–2 mm in diameter. BLI, ^18^F-FDG PET and necropsy analyses showed relevant PM in all animals.

**Conclusions:**

The USN together with the PM rat model are suitable for robust and species-specific preclinical pharmacological studies regarding intraperitoneal delivery of pressurized aerosolized drugs and cancer research.

## Introduction

One decade ago, intraperitoneal pressurized aerosol chemotherapy (PIPAC) was clinically introduced as a new technology to deliver intraperitoneal (IP) chemotherapy for palliation of patients with unresectable peritoneal metastases (PM). Due to encouraging clinical results, it was rapidly distributed world-wide with more than 11,000 reported applications until the end of the year 2018 [1]. The efficacy of PIPAC benefits from various physical phenomena [2–4]. Firstly, the drug delivered as an aerosol should lead to an improved spatial distribution pattern compared to that of liquid IP administration. Secondly, both the increased capnoperitoneal pressure and the increased drug concentration of the aerosol enhance drug concentration and penetration depth into PM tissue [5]. Another pharmacological benefit is that the capnoperitoneal pressure decreases the splanchnic blood flow, thus leading to a reduced systemic drug absorption [6]. Therefore, a reduction of detrimental side effects of anti-cancer drugs is expected.

However, there is still limited evidence on the extent to which the current technique and the therapeutic parameters used for PIPAC have pharmacokinetic/oncological advantages compared to systemic or liquid IP chemotherapy. To get a better insight and understanding of this promising technology, there is an urgent need for preclinical research on pressurized intraperitoneal drug aerosol therapy in representative and reproducible animal models. Due to the considerable geometric dimensions of the currently used technology (e.g.: nozzle diameter of 12 mm), preclinical research should ideally be performed in large animals (e.g.: pigs). But research on large animals is expensive and relevant PM models are missing.

The suitability of an ovarian PM rat model to study pharmacokinetics and anti-tumor efficacy of PIPAC with the nebulizer technology and operative setup as applied to humans has been recently reported [7, 8]. However, this technology developed for human use presents some technical features which limit its appropriate use in small animal models. A minimum of two balloon trocars (5 mm and 12 mm) are needed for access to the abdominal cavity, which take up 30 to 40 mL of volume of the abdominal cavity. Together with the volume of IP injected liquid (approx. 20 to 30 mL), half of the total volume of the rat capnoperitoneum (approx. 130 mL) is filled out [7]. In addition to a large wound to body size ratio for trocar positioning, the characteristics of the generated aerosol and its spatial distribution depend strongly on the spraying distance [9], the entry point and the entrance angle of the nozzle into the abdominal cavity. At low spraying distances (< 6 cm), as expected in the rat model, more than 98 vol.-% of the aerosolized drug impacts directly with the peritoneum in the vicinity of the nozzle and form a liquid film. Moreover, the aerosol generation process of human PIPAC technology suffers also from a significant initiation and ending phase for which, a minimum of 20 to 30 mL liquid drug must be aerosolized to obtain a minimal length of a steady-state aerosol generation [10, 11]. This is furthermore in conflict with international guidelines about good practice for the IP administration of liquids in animals, which specify a maximum drug administration of 5 mL in 250 g weighing rats [12].

With regard to future preclinical small animal routine research on pressurized intraperitoneal aerosolized drug therapy, this study deals with the development and technical characterization of a modified ultrasound nebulizer (USN) device and an operative setup to refine the IP administration of aerosolized small drug volumes via a down-scaled minimal-invasive operative setup suitable for small animal research in accordance with the rules for animal welfare regulations.

Due to the technical operating parameters of the developed nebulizing procedure, a rodent model, most specifically in rat, for size issues, was required. For this reason, and because only a very limited number of orthotopic rat models of colorectal cancer are currently established [13], a new orthotopic colorectal PM rat model was developed. In pilot experiments, detectability and quantification of early PM with bioluminescence (BLI) and 2-deoxy-2-[fluorine-18]fluoro-D-glucose positron emission tomography imaging (^18^F-FDG-PET) was explored. Finally, the practicability, technical handling and tolerance of repetitive pressurized intraperitoneal aerosol applications with the USN and the down-scaled minimal-invasive operative setup was studied.

## Material and methods

### Technical design, ex-vivo optimization and technical validation of the ultrasonic nebulizer (USN)

#### Design of the USN and its corresponding setup for pressurized intraperitoneal aerosol supply

The extra-corporal aerosol generation based on a commercial ultrasonic nebulizer (Fisoneb, Fisons/Medix Electronic/Karapharm, Marseille, France) that was technically modified by FVM Technologies & Consulting (Palaiseau, France). For the operation of the device with CO_2_ instead of air, the internal ventilator of the device was replaced by a pipe connected to compressed CO_2_ via a high-pressure regulator to an adjustable flow-rotameter. To obtain a higher drug aerosol output, the electrical power of the 12 VDC power supply was increased from 2 W to 6 W without modification of the ultrasonic probe frequency of 110 kHz.

Aerosol transportation from the outlet of the USN to the inlet to the rat was realized via a silicon tube with an inner diameter of 5 mm and a total length of 305 mm and a catheter (Surflow® I.V. Catheter 14G, ID SR + OX1464C1, Terumo) with an inner diameter of 1.73 mm. Preliminary ex-vivo analyses showed that condensation phenomena due to temperature differences between aerosol stream and the environment led to liquid film formation from the aerosol on the inner tube surface and thus to dripping at the outlet opening of the tube. To minimize particle loss by aerosol condensation and to prevent disturbance of the aerosol flow, the silicon tube was introduced into a heating pipe regulated to 40 °C (FVM Electronic Company, Palaiseau, France).

#### Ex-vivo optimization of the operating parameters of the USN by ^99m^TC scintigraphy

A biocompatible capnoperitoneal pressure of 8 to 10 mmHg in rats has been reported already previously [7, 8]. At that pressure the spatial geometry and the volume of the rat capnoperitoneum was determined by means of breathing-triggered computer tomography volumetry (Micro-CT SkyScan 1278, Bruker, Billeric, USA) in five healthy male Wistar rats (Charles River, France) of 250 to 350 g body weight. A median volume of the rat capnoperitonea at a pressure of 8 to 10 mmHg of 130 ml ± 10 ml was observed [14]. Based on these data, we designed artificial rat capnoperitonea phantoms by using cylindrical polyethylene terephthalate cans that were internally coated with a 1 to 2 mm thick agar layer and had an approximately similar geometry dimension of 4.0 × 10.5 cm as the in-vivo rat capnoperitoneum. Optimization of the operating parameters to maximize IP aerosol deposition were determined by real time scintigraphy (Gamma Imager, Biospace, Mesures, France with IRIS Software Aries Nucleaire, France). For this purpose, 99^m^Tc-labelled human plasma albumin scintigraphic studies was chosen due to its low absorption by both lymphatic and blood capillaries in view of the later in-vivo studies.

The freeze-dried radiopharmaceutical was dissolved under vacuum into 3 mL saline. After 5 min, 2 GBq of ^99m^Tc sodium pertechnetate (Cis bio, France) were added. After further 5 min incubation at room temperature, the resulting solution was used to load the liquid chamber of the USN. At a CO_2_ flow of 2 L/min, an optimum of ^99m^Tc-labelled human plasma albumin aerosol deposition of 30 MBq/1.5 ml inside the rat capnoperitoneum phantoms was determined by real time scintigraphy.

#### Ex-vivo granulometric analyses of the USN aerosol

Granulometric aerosol analyses were performed by means of laser diffraction spectrometry (LDS). Therefore, a laser diffraction spectrometer (HELOS/KR-H2487, Sympatec GmbH, Germany) according to ISO 13320:2009 [15] was operated for determining volume-weighted droplet size distribution over a size range from 0.5–175 μm [16]. Aerosol characterization was performed at a distance of 5 mm from the aerosol outlet and the laser beam operated as reported in prior studies [10, 11]. For the purpose of the analysis, the USN was operated with a 0.9 wt.-% aqueous sodium chloride solution (B. Braun Melsungen AG, Melsungen, Germany). The time resolution was set to 3 s at a total measurement time of 10 min.

### Pressurized intraperitoneal aerosol supply studies and orthotopic PM model in rats

#### USN and corresponding setup for pressurized intraperitoneal aerosol supply in rats

**Fig. 1:**
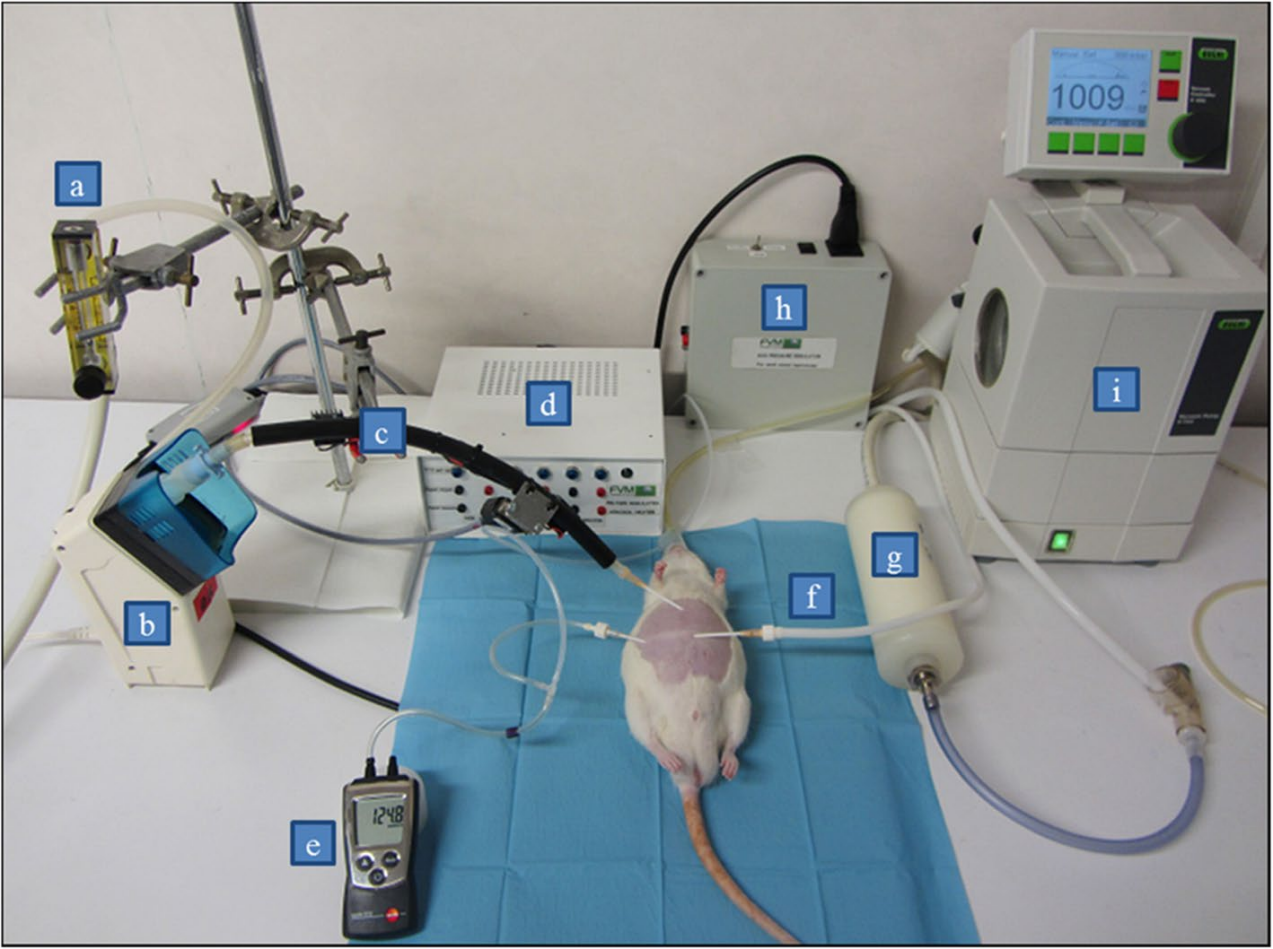
Experimental setup for minimally invasive pressurized intraperitoneal drug aerosol supply in a rat placed in supine position on a 37 °C heating pad; a = adjustable CO_2_ flow meter; b = modified ultrasonic nebulizer (USN); c = aerosol heating tube with integrated aerosol inlet tube (40 °C); d = adjustable aerosol tube heating device; e = capnoperitoneal pressure monitor; f = aerosol outlet tube; g = high efficiency particulate air (HEPA) filter capsule; h = electronic pressure regulator device; i = aerosol evacuation pump

The setup for pressurized intraperitoneal aerosol supply designed for in-vivo experiments is shown in Fig. 1 and comprises an extra-cavitary aerosol generation based on a modified ultrasonic nebulizer (USN), heated tubing for aerosol transportation and indwelling vein cannulas for aerosol flow supply and outlet to/from the abdominal cavity of the rats.

By means of a simple puncture into the abdominal cavity, a catheter (Surflow® I.V. Catheter 14G, ID SR + OX1464C1, Terumo) with an inner diameter of 1.73 mm was used for aerosol supply into the rat’s abdomen. Correct puncture of the abdominal cavity was checked by aspiration and saline sip test. The capnoperitoneum was then established and analogous to the insertion of the aerosol inlet catheter, two further catheters (Surflow® I.V. Catheter 14G, ID SR + OX1464C1, Terumo) were inserted into the abdominal cavity of the rat. One catheter was connected to a HEPA filter capsule (ID: 1602051, TSI Inc., Shoreview, USA) to separate any particulate contamination from the outlet aerosol flow of the abdomen. Immediately before the inlet of the absolute filter, a pressure control unit (FVM Technologies & Consulting, Palaiseau, France) was placed to adjust and regulate the IP pressure to 8 to 10 mmHg in order to set a stable and reproducible capnoperitonea. The third catheter (Surflow® I.V. Catheter 16G, ID: SR + OX1651C1, Terumo) was laterally positioned into the abdomen to monitor the actual intraperitoneal CO_2_ pressure (Testo 510 differential pressure meter, Testo S.a.r.l, Forbach, France). The three catheters were anatomically positioned similarly in all experiments.

#### Orthotopic human colon cancer peritoneal metastasis (PM) model

For the development of an orthotopic peritoneal metastasis (PM) model of colorectal cancer, 12 male immunosuppressed 250 to 300 g RNU rats (Crl: NIH-Foxn1rnu, Charles River, France) previously irradiated at 5 Gy with Co-60, received an IP administration of HCT116-Luc2 human colon cancer cells (Perkin Elmer, France) that express stable luciferase gene for bioluminescence imaging. HCT116-Luc2 cells were cultured at 37 °C and 5% CO_2_ in Mc Coy’s 5a (modified) medium (Gibco® 26,600–023, Thermo Fischer Scientific) supplemented with 10% fetal bovine serum (Gibco®) and 1% Penicillin-Streptomycin (Gibco®). Each rat received 1 × 10^7^ HCT116-Luc2 cells, suspended in 2 mL phosphate buffered saline (Gibco®) under gas anesthesia (2% air/isoflurane mixture, Iso-Vet, Piramal Healthcare) IP. To improve the homogeneity of metastatic patterns, two separate IP injections laterally into the abdominal cavity of 1 mL each containing 5 × 10^6^ cells were performed.

#### Aerosol and liquid IP deposition and spatial distribution studies in rats

Single photon emission computed tomography (SPECT) imaging with IP ^99m^Tc-labelled human plasma albumin administration, analog radiopharmaceutical preparation as described above was performed in a total 15 healthy male Wistar rats (Charles River, France) of 250 to 350 g body weight.

First, the radiopharmaceutical was administered IP in five rats under 2% isoflurane anesthesia using the USN for pressurized intraperitoneal aerosol supply (Group 1). The drug chamber of the USN was loaded with 185 MBq of ^99m^Tc labelled human plasma albumin in NaCl 0.9 wt.-% to a total volume of 10 ml. The drug was then nebulized during 10 min into a stable capnoperitoneum of 8 to 10 mmHg which was maintained for another 20 min. Whole body counting (Dose Calibrator, Capintec, Inc., Florham Park, NJ, USA) showed decay corrected median dose of 30 ± 0.2 MBq of radiopharmaceutical deposited IP in the animals corresponding to 15 wt.-% (1.5 mL) of the total 10 ml ^99m^Tc labelled human plasma albumin loaded in the liquid drug chamber of the USN. To assess the effect of the 8 to 10 mmHg capnoperitoneum on the spatial distribution pattern, simple liquid IP injection of equivalent doses/volume (30 MBq ^99m^Tc labelled human plasma albumin in 1.5 ml) were performed either with (Group 2) or without (Group 3) capnoperitoneum. The chronology of the experiments performed was as follows:

- *Group 1; (n = 5):* IP drug aerosol supply with the USN at a CO_2_ flow rate of 2 L/min for 10 min (IP deposited dose/volume of 30 MBq/1.5 mL) into a constant capnoperitoneum of 8 to 10 mmHg pressure which was maintained for another 20 min after the end of nebulizing under anesthesia
- *Group 2; (n = 5):* manual liquid IP injection by means of a small-caliber puncture syringe of 30 MBq/1.5 mL into a constant capnoperitoneum of 8 to 10 mmHg pressure which was maintained for 30 min under anesthesia
- *Group 3; (n = 5):* manual liquid IP injection by means of a small-caliber puncture syringe of 30 MBq/1.5 ml without capnoperitoneum followed by 30 min of anesthesia

SPECT imaging started 30 min after the administration of the radiopharmaceutical without capnoperitoneum in all three groups and was performed in a dedicated, multiplexed multi-pinhole small animal SPECT/CT imaging device (NanoSPECT/CT, Mediso, Budapest, Hungary). The system was equipped with 4 collimators (nine pinholes, aperture 1.5 mm). The detection energy window for ^99m^Tc was set to 140 keV ± 10%. Helical scanning mode was used and 24 projections of 30 s were acquired resulting in 21 min scans. During the scan, the rats were kept under 2.0% isoflurane (Iso-Vet, Piramal Healthcare, UK) anesthesia in air carrier at 0.5 L/min and maintained at 37 °C. The imaged data were reconstructed (HiSPECT NG software. Scivis GmbH, Göttingen, Germany) and analyzed with image analysis software (InVivo Scope, Bioscan Inc., USA).

For quantitative spatial distribution analysis ^99m^Tc labelled human plasma albumin, SPECT/CT full imaging along the main body axis (250 images of 92 × 92 pixels) were stored in Digital Imaging and Communications in Medicine (DICOM®) files. A custom computer program (source code available on GitHub at https://github.com/grezniczek/SPECTalyzer) was used to process these files. First, the 100 consecutive slices that contain the highest combined signal intensities were identified. Then, data points were ordered from highest (top) to lowest signal value and the number of data points (from the top) were counted that were needed for their cumulative signal to exceed a given percentage (30%) of the total cumulative signal in the 100 slices. This number, the TVE30 (top voxels exceeding 30%), is represented as the fraction of the total number of data points. Thus, higher TVE30 values correspond to a more uniform distribution. SigmaPlot14 (Systat Software Inc., San Jose, CA) was used for statistical analysis. The Shapiro-Wilk test was used to assert normal distribution of experimental data. Paired t-test was used to compare within experimental groups. All reported *p*-values are two-tailed.

#### Monitoring of PM induction and growth

Monitoring of tumor proliferation was performed in-vivo by bioluminescence imaging (BLI), 2-deoxy-2-[fluorine-18]fluoro-D-glucose PET imaging (^18^F-FDG-PET) and necropsy. Prior bio-luminescence imaging (Ivis® Lumina II, Perkin Elmer, France), rats received an injection of 40 mg Luciferin (Perkin Elmer, France) 4 min before induction of anesthesia (2% air/isoflurane mixture (Iso-Vet, Piramal Healthcare)). The rodents were imaged once a week over 3 weeks at both posterior and anterior faces. Regions of interest were drawn on the anterior and posterior surface of animals in the abdomen and quantified via the bioluminescence signal (Living Image 4.4, Perkin Elmer, France) to document the evolution of PM. Results were expressed in numbers of emitted photons per second.

Five animals were selected on the basis of BLI to undergo 18F-FDG-PET examination on day 17 (D17) post HCT116-Luc2 cell injection. For PET, the radiopharmaceutical fluorine-18-fluordeoxyglucose (^18^F-FDG, AAA, France) with an activity of approximately 35 MBq was injected into the tail vein of the rats, which had free access to fresh water but not to food for 12 h before the examination. After 1 hour of tracer distribution in the body, animals were placed in the bed of a micro-PET scanner (eXplore VISTA, Sedecal, Spain). Therefore, the rats were anesthetized with an air/isoflurane mixture (5% for induction and 2% in steady state; Iso-Vet, Piramal Healthcare) and maintained at 37 °C during the examination. For a whole-body image, a sequence of 3 scans with 3 bed positions was acquired. Each bed position was scanned for 15 min that cumulates in a total scanning time of 45 min. Radionuclide decay was automatically corrected between each bed position. The energy window was set to 250–700 keV. Images were reconstructed and visualized by MMWKS Image Software (4.7 Build 452). 3D FORE/2D OSEM algorithms were used for image reconstruction.

Thirty days post induction of the human colon cancer cells, all rats were sacrificed by an overdose of anesthesia and then autopsied to characterize metastases and to compare their anatomical locations to foci observed by BLI and 18F-FDG-PET.

#### Feasibility of repetitive applications of pressurized intraperitoneal aerosol

The technical feasibility and handling of USN and the corresponding minimally invasive operative setup for repeated intraperitoneal pressurized aerosol administration in rats were explored on day 7 (D7), day 14 (D14), day 21 (D21), and day 28 (D28) after intraperitoneal instillation of tumor cells immediately following BLI imaging (see Fig. 1). The drug chamber of the USN was loaded with 10 ml 0.9 wt.-% sterile aqueous sodium chloride solution for aerosol generation.

## Results

### Performance characteristics of the ultrasonic nebulizer (USN) and ex-vivo optimization

**Fig. 2:**
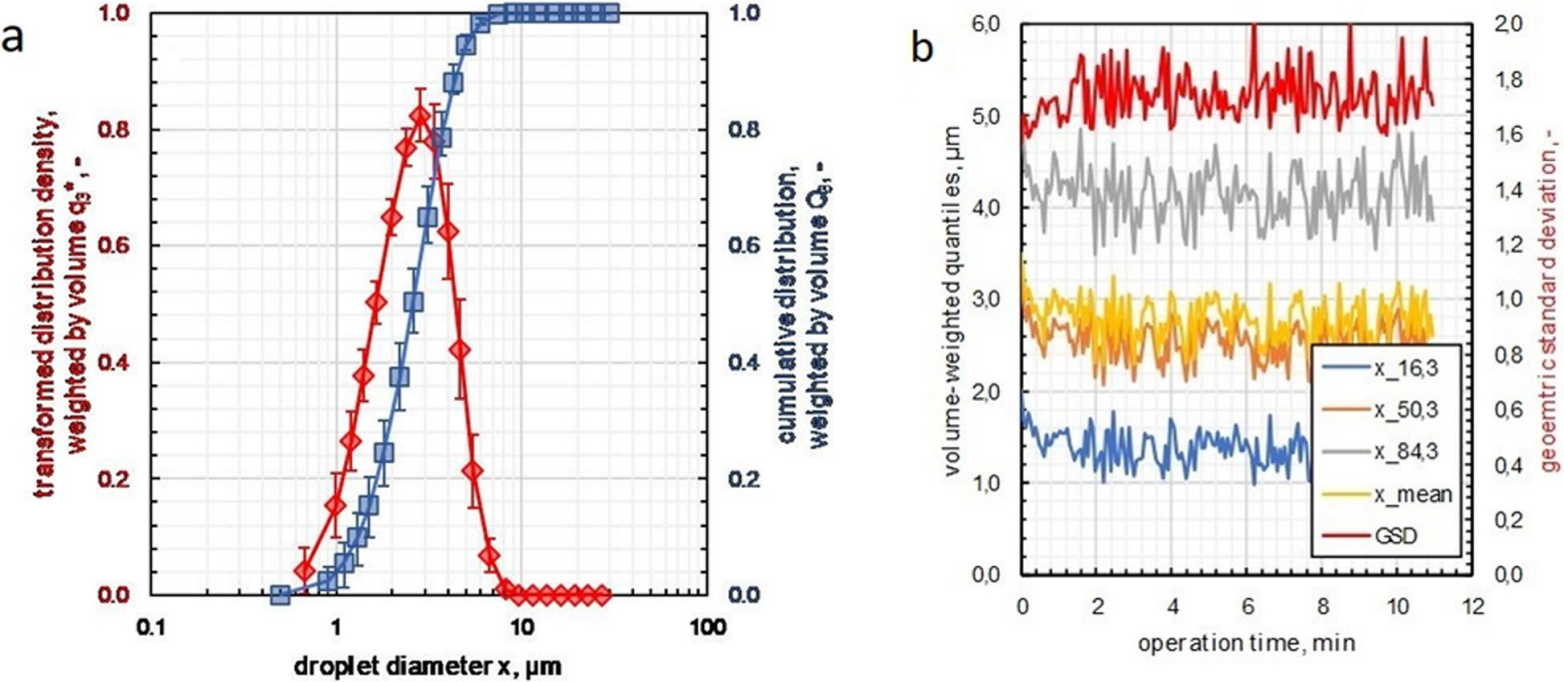
Laser diffraction spectrometry: Mean volume-weighted particle size distribution of the aerosol at the outlet of the nebulizer (**a**) and volume weighted quantiles of the aerosol over time (**b**); x_16,3_ = 16% quantile, x_50,3_ = 50% quantile or median diameter, x_84,3_ = 84% quantile, GSD = geometric standard deviation

In order to avoid condensation of the aerosol in the aerosol outlet pipe, a preheating time of the outer heating pipe of 12 minutes was necessary to reach its constant operating temperature of 40 °C. The temperature of the outflowing aerosol at a CO_2_ flow of 2 L/min at the catheter tip was 37 °C. The results of the granulometric characterization of the USN aerosol with these operating parameters are summarized in Fig. 2.

According to Fig. 2 a, the USN provides a polydisperse, monomodal aerosol with a volume-weighted median diameter of x_50,3_ = 2.59 μm ± 0.2 μm and a geometric standard deviation of σ_g_ = 1.73 ± 0.05. Furthermore, USN shows neither an initial phase nor a shut-down phase in aerosol generation as it can be observed in Fig. 2 b. The aerosol of the USN is thus ready for use immediately after initiation of generation.

The optimal performance of the USN was determined at a CO_2_ flow rate of 2 L/min. This resulted to a drug-aerosol flow in the USN of 1.0 mL/min. After 10 min of continuous nebulization, SPECT studies on the rat phantoms showed a total of 30 MBq of ^99m^Tc labelled human plasma albumin deposition corresponding to a deposition rate of 15 wt%.

### In-vivo quantification of intraperitoneal radiopharmaceutical deposition and distribution

The technical handling of the modified USN and the corresponding operational setup proved to be simple and intuitive. All experiments were performed without any technical problem. A stable capnoperitoneum of 8 to 10 mmHg could be established within 10 to 15 s after a stepwise increase of the CO_2_ flow to its maximum of 2 L/min. After a stable capnoperitoneum was established, the tightness of the capnoperitoneum was monitored. No CO_2_ leaking at the catheters or elsewhere in the setup was observed during any experiment. No manual readjustment of the CO_2_ flow was necessary during the further course of the USN experiments. At a capnoperitoneal pressure of 8 to 10 mmHg, a median capnoperitoneal volume of 130 +/− 10 mL was determined. With a CO_2_ flow rate of 2 L/min, a mean capnoperitoneal residence time of the aerosol inside the abdomen was calculated to be 3.9 s. All animals, showed a well toleration of the capnoperitoneum and no signs of cardio-respiratory restriction were observed.

After the USN experiments (Group 1), whole body dosimetry (Dose Calibrator, Capintec, Inc., Florham Park, NJ, USA) showed decay corrected a median dose of 30 ± 0.2 MBq of radiopharmaceutical delivered intraperitoneally per animal. In-vivo IP aerosol deposition proved to be similar to that observed in the ex-vivo rat phantom experiments.

**Fig. 3:**
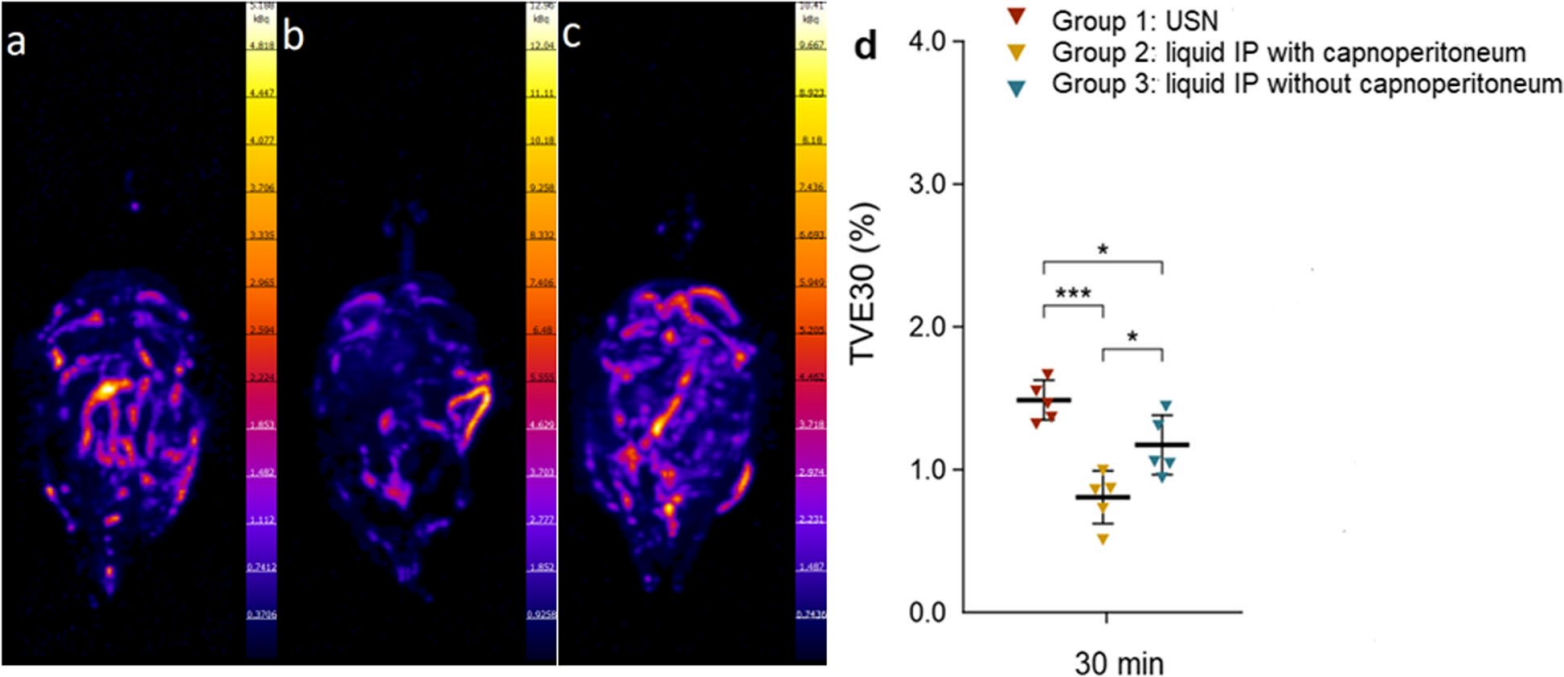
SPECT/CT analysis of the intraabdominal drug distribution pattern of ^99m^Tc labelled human plasma albumin. Left panel: Representative SPECT-CT scans (**a, b, c**) of rates in anterior-posterior maximum intensity projection (MIP) after 30 min; a = Group 3 (intraperitoneal injection without capnoperitoneum); b = Group 2 (liquid intraperitoneal injection with capnoperitoneum); c = Group 1 (USN with capnoperitoneum). Right panel (**d**): drug distribution represented by number of top voxels needed to exceed 30% (TVE30) of the cumulative signal in the measured volume, the higher TVE30 the higher the uniformity of drug distribution. Triangles represent individual rats (*n* = 5), thick horizontal lines are means, error bars represent standard deviations; Statistical significance (one-way ANOVA) is indicated by * (*p* < 0.05), *** (*p* < 0.001). 30 min = after the start of application of the radiopharmaceutical

Spatial distribution analyses by SPECT-CT imaging (Fig. 3) revealed that 30 min after the administration of the radiopharmaceutical, distribution was most homogeneously in the USN group (Group 1 vs. Group 2: *p* < 0.05; Group 1 vs. Group 3: *p* < 0.001), as measured by TVE30 (see Materials and Methods for details; higher values signify a more homogeneous distribution). Comparing the two groups of liquid intraperitoneal injection, a significantly worse spatial distribution pattern was found for the group with established capnoperitoneum (Group 2 vs. Group 3: *p* < 0.05).

### Monitoring of induction, growth and dissemination of PM

**Fig. 4:**
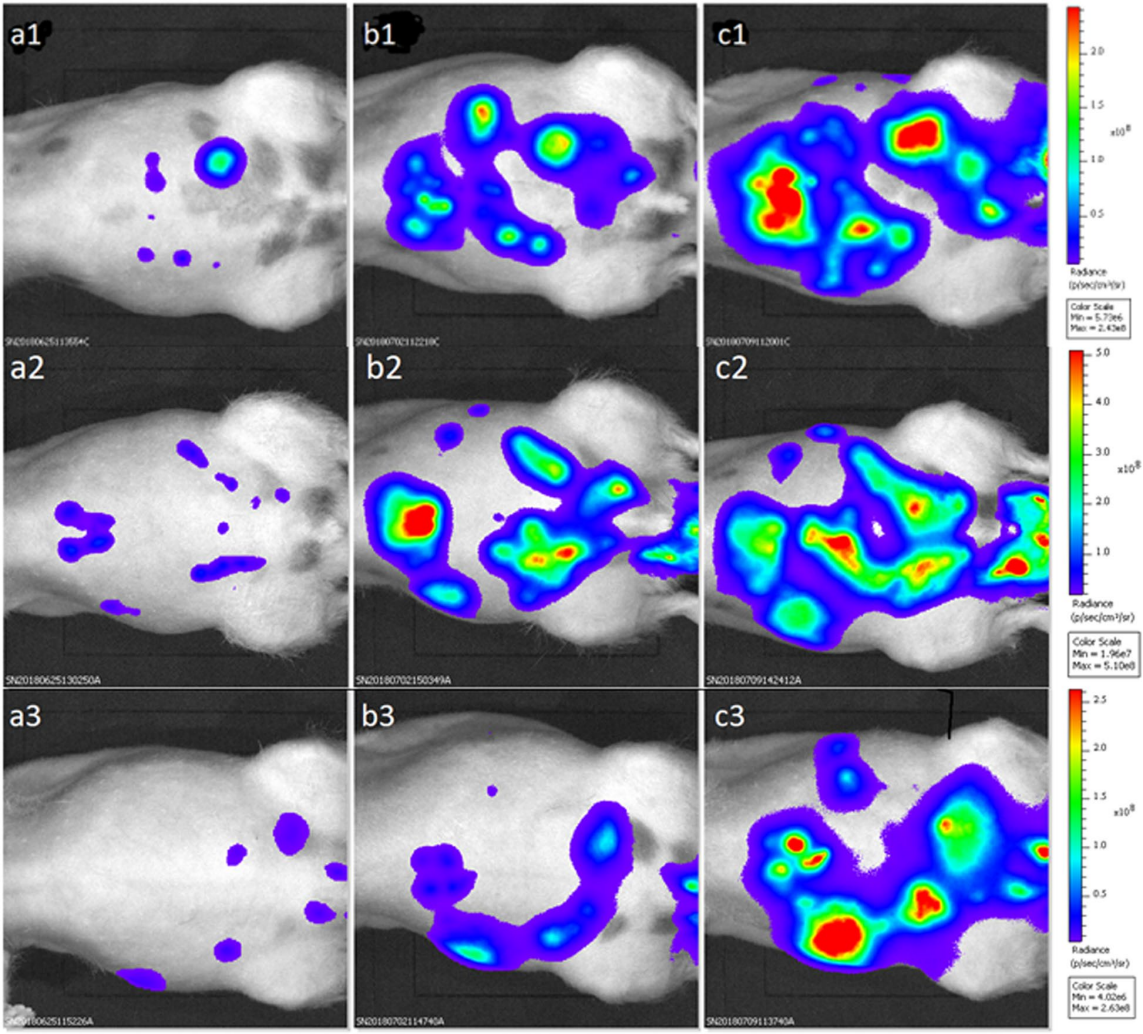
Bioluminescence imaging: Monitoring of tumor growth after 7, 14 and 21 days (D) post induction of xenografts. All animals shown as ventral view. Upper panel rat R75: a1 = D7; b1 = D14; c1 = D21. Panel in the middle rat R77: a2 = D7; b2 = D14; c2 = D21. Lower panel rat R76: a3 = D7; b3 = D14; c3 = D21

Tumor growth was characterized in-vivo by BLI and ^18^F-FDG-PET, and at necropsy. Figure 4 provides representative images of BLI to study tumor growth and tumor dissemination over time in three rats (R75, R77, R79). The animals underwent BLI imaging at day 7 (D7), day 14 (D14), day 21 (D21) and day 28 (D28) after intraperitoneal tumor cell instillation.

**Fig. 5:**
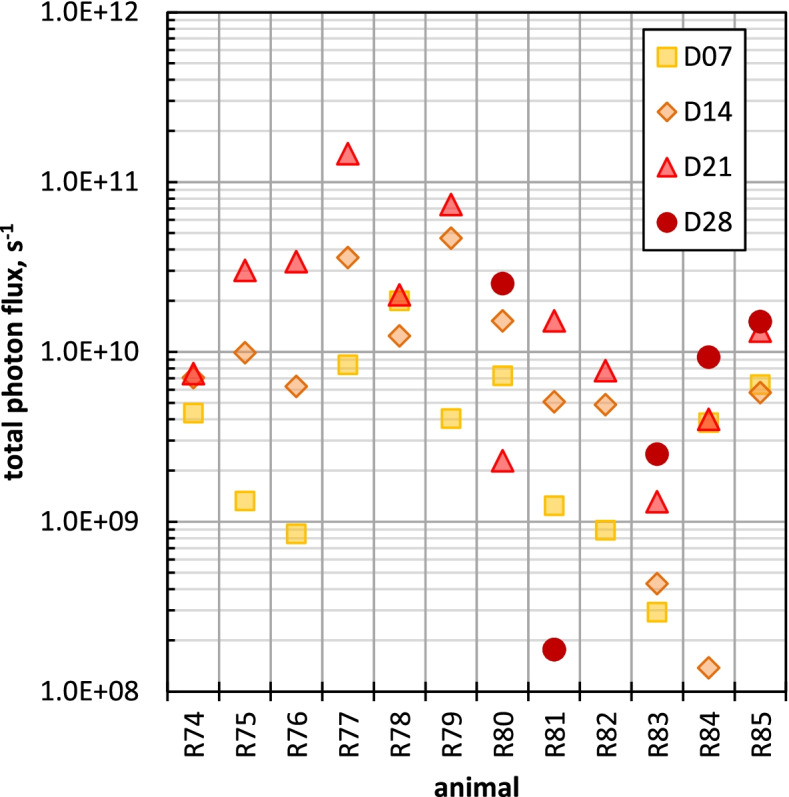
Bioluminescence Imaging: Photon flux (signal intensity) over time after 7 days (D7), 14 days (D14), 21 days (D14) and 28 days (D28) post induction of the xenografts. The animals R80 to R85 additionally underwent examination by bioluminescence at day 28 (D28)

BLI results of all animals at day 7 (D7), day 14 (D14), day 21 (D21) and day 28 (D28) after intraperitoneal tumor cell instillation are summarized in Fig. 5.

**Fig. 6:**
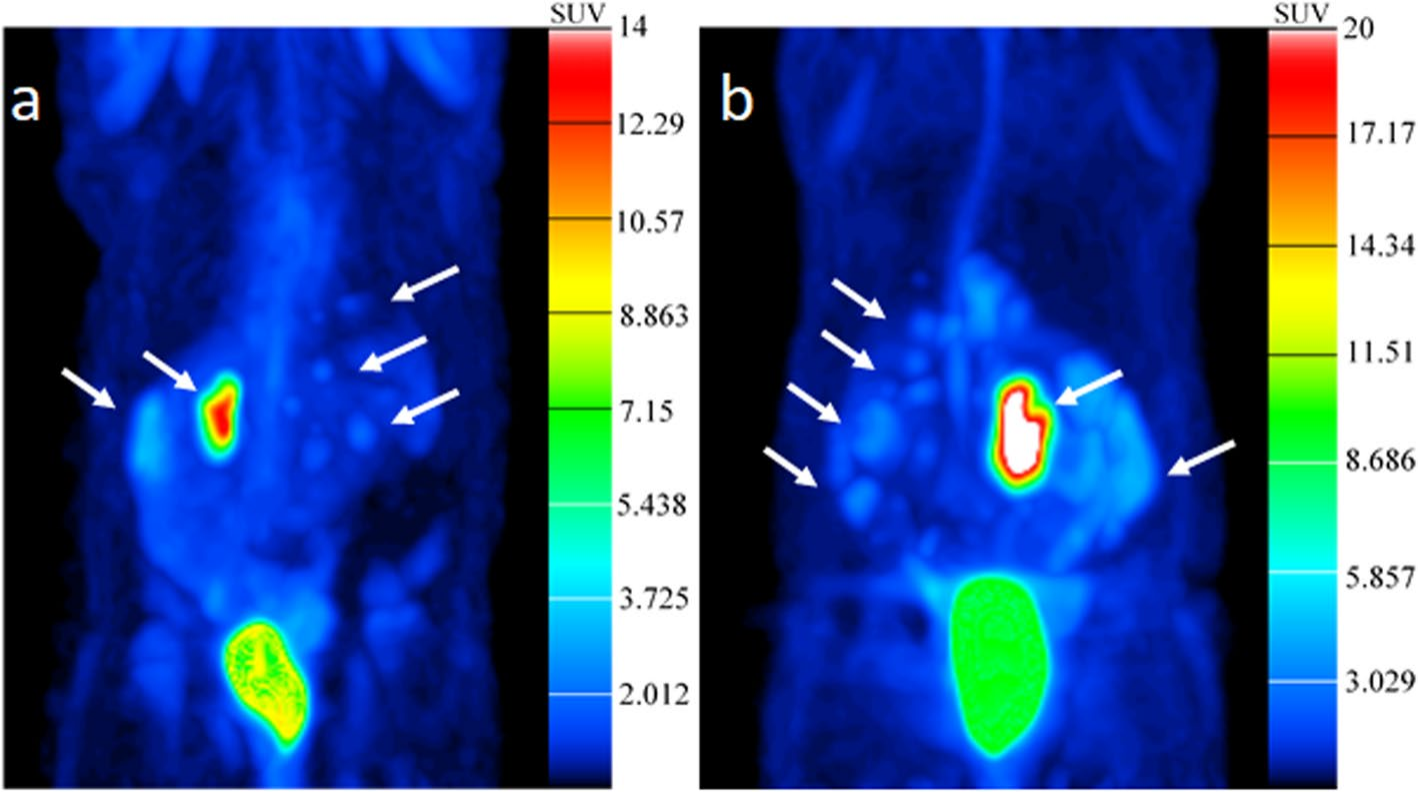
^18^F-FDG-PET imaging on day 17 (D17) post HCT116-Luc2 cells injection for rat R76 (**a**) and rat R77 (**b**); white arrows indicate detected tumor foci. The colored scale corresponds to SUV values. MIP (Maximum Intensity Projection) visualization. Curvature Flow Filtering (Iter 10, Time Step 0.2). Area with high intensity inside the bladder was removed

A certain spatio-temporal inhomogeneity of the BLI signal intensity was observed between the animals. Nevertheless, BLI findings indicated a sufficient and relevant induction of PM in all animals with a diffuse intraperitoneal distribution pattern. However, raw photon flux data measures for PM detection and quantification should be interpreted with care. Technical limitations of BLI, like differences of photon absorbance of surrounding tissue, nodules located deep inside the peritoneal cavity, will not be delineated due to limited depth penetration of the light, a moderate spatial resolution and possible signal overexposure in case of intense tumor growth must be taken into account. Data derived from in-vivo PM imaging, mainly in mice models, report that the tumor load may be underestimated when using BLI whereas ^18^F-FDG PET is more sensitive, especially for the detection of early PM and nodules located deeper into the abdominal cavity [17–20]. In contrast to BLI, intestine movements can be eliminated computationally using positron emission tomography (PET) by synchronization with respiration signals. Therefore, to further evaluate the accuracy of PM detection and quantification in our PM rat model, five animals (R76, R77, R80, R84 and R85) were selected on the basis of BLI to undergo ^18^F-FDG-PET examination on day 17 (D17) post HCT116-Luc2 cell injection. Representative ^18^F-FDG-PET images of two rats (R076, R077) are shown in Fig. 6.

^18^F-FDG-PET could be performed successfully in all animals. According to Fig. 6, the tumor cells of the human colon cancer PM model show a well uptake and accumulation of ^18^F-FDG. Compared to BLI, ^18^F-FDG-PET detected even smaller PM nodules measuring only 1 to 2 mm in size.

**Fig. 7:**
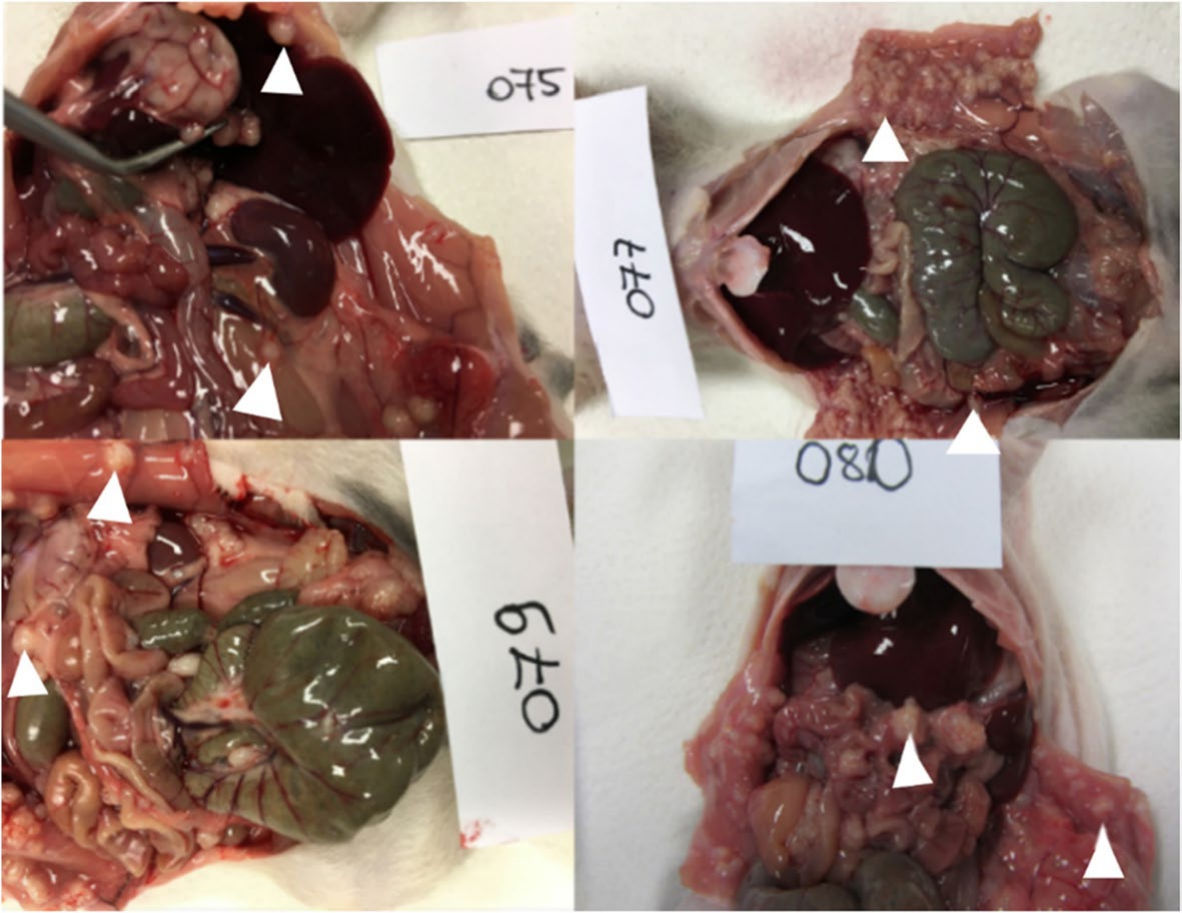
Post-mortem view of the tumor manifestation and distribution of four animals (R75, R77, R79, R80) after intraperitoneal injection of 1 × 10^7^ HCT116-Luc2 cells; white triangles indicate the observed tumor nodules on the parietal and visceral peritoneum

Thirty days (D30) after HCT116-Luc2 cell inoculation, all animals underwent necropsy. Representative photographic images of the peritoneal tumor involvement of four rats (R75, R77, R79, R80) are shown in Fig. 7.

Similar to BLI and ^18^F-FDG-PET, necropsy showed differences in tumor growth and dissemination between the different animals. However, a macroscopic and clinically relevant PM induction, with tumor spread on the visceral and parietal peritoneum, was achieved in all animals.

## Discussion

All over the world, pressurized intraperitoneal aerosol chemotherapy (PIPAC) is increasingly employed in clinical practice to manage patients suffering from end-stage PM of gynecological and non-gynecological origins [1]. However, firm preclinical or clinical evidence demonstrating that intraperitoneal pressurized aerosol treatment would provide an oncologic advantage over conventional treatments is still limited. More recently, PIPAC technology, as employed for in human use, has been reported as also technically feasible in rats [7]. First preclinical data about an increased anti-tumor effect compared to simple liquid intraperitoneal drug instillation in an ovarian cancer rat PM model are encouraging [8]. However, the considerable geometric dimensions and the limitations of the technology used for humans use restrict its reasonable application for basic small animal research. Finally, the use of not optimized technology to small animal research harbors also the risk that any therapeutic potential of this innovative therapeutic approach could be flawed or even underestimated.

To suit the technical and animal welfare needs for future small animal research on intraperitoneal pressurized aerosol drug application, a minimal invasive, reusable operative setup was developed and validated. Core piece of the setup is a modified ultrasonic nebulizer (USN) for extra-cavitary aerosol generation. In combination with measures for aerosol conditioning, this setup provides a physically defined and stable aerosol with quasi negligible initial aerosol formation phase. Optimal performance of the USN in rats was found for an aerosol flow rate of 2 L/min and a drug flow rate of 1 mL/min, whereof approx. 15 wt.-% of aerosolized substance is deposited onto the rat peritoneum during a mean capnoperitoneal residence time of 3.9 s (capnoperitoneal volume of approx. 130 mL at a capnoperitoneal pressure of 8 to 10 mmHg). Granulometric analyses showed that the USN delivers a polydisperse, monomodal aerosol that, in comparison to the conventional nebulization technology for human use [10], has a droplet size which is i) 10 times lower, ii) less affected by gravity and inertia, iii) less dependent on spraying distance as well as spraying angle to the peritoneum and iv) should thus be accompanied with an even more homogeneous spatial drug deposition. Single photon emission computed tomography (SPECT/CT) showed that drug application with the USN lead to a significant more homogenous drug distribution where as pure liquid injection and liquid injection into an established capnoperitoneum showed a worse spatial distribution pattern.

Previous data about the human nozzle technology report that a homogeneous intraperitoneal drug distribution can be expected with a volume-weighted median diameter of the aerosol droplets of < 1.2 μm [10]. It is evident that the USN, with its droplet size nearly ten times smaller than human nebulizer technology, results in a more homogeneous spatial distribution pattern of the aerosol. Nevertheless, a direct comparison between the USN and the human nebulizer technology currently in use is not practical because human technology cannot be operated at such low volumes as used with the USN. Of note, we would like to emphasize that the developed USN is not intended to replace the current nebulization technology used in humans. Instead, with the appropriate minimally invasive surgical setup, the USN has several advantages in small animal use that can facilitate and optimize preclinical research on pressurized intraperitoneal aerosol delivery. However, should future studies demonstrate potentially superior anti-tumor efficacy compared to other modes of delivery, or any other substantial advantages not available with other techniques, then, and after establishment of technical feasibility in large animal models, the USN could be considered for human application as well.

The minimally invasive approach is a technical refinement to improve animal welfare, as no surgical intervention is required for intraperitoneal aerosol administration. This fact is even more important since it is currently assumed, that PIPAC must be repeated at least three times at three to six-week intervals. The optimal time interval between PIPAC administrations has not yet been studied and thus remains arbitrary. With regard to small animal studies of significantly shorter therapy intervals, interference with wounds and wound healing disorders are not expected with USN technology.

Another advantage of extra-corporeal aerosol generation is the fact that the USN technology can be operated with smaller volumes (< 5 ml) than the nebulizer technology currently used in humans (> 30 ml). Thus, USN technology is consistent with good practice in small animal research which allows a maximum intraperitoneal administration of 5 ml of fluid in animals weighing 250 g [12]. Furthermore, the USN technology offers the possibility to evaluate the influence of higher drug concentration at constant drug dose by reducing the volume of the carrier solutions to be nebulized. This is only possible to a limited extent with the current monocomponent nebulizer technology as used in humans, since a minimum volume of 30 ml is required. Higher drug concentrations can therefore only be achieved by simultaneously increasing the drug dose. Finally, the USN can also be used to investigate the potential therapeutic impact of the duration of the active nebulization process (aerosol phase), as an active nebulization of up to 30 minutes (total intraperitoneal volume < 5 ml) will still be in line with the standards of good practice in small animal research.

The repeated use of the USN nebulizer and its minimally invasive aerosol delivery setup was easy to handle, reliable, and the amount of aerosol delivered intraperitoneally was constant in each animal. Because the USN is autoclavable and only a few parts of the operative setup such as the catheter parts are inexpensive disposable items, the developed system is reusable and its operation inexpensive.

To satisfy the need of a small animal model for pre-clinical further anti-cancer research with the USN, a colorectal cancer PM rat model was established. We establish a HCT116-Luc2 human colon cancer cells PM model in immunosuppressed RNU rats. BLI and ^18^F-FDG-PET imaging revealed the induction of a relevant PM in all animals, however demonstrating a certain spatio-temporal inhomogeneity of tumor growth and distribution between the animals.

Pilot experiments of in-vivo PM explorations by BLI and ^18^F-FDG-PET showed that each technology represents a valuable tool. BLI appears to be more sensitive than PET to detect early metastases and has the advantage to be non-irradiating towards tumors at the time examinations must be repeated. However, it suffers from an intrinsic low spatial resolution which is additionally mitigated by movements of intestines due to peristaltism and respiration while tissue absorption results in decreased accuracy for quantitation in rats [20]. ^18^F-FDG-FDG-PET resulting in intense fixation of the tracer in HCT116 tumor cells allows millimetric resolution for imaging metastatic dissemination with reliable quantitation of the aerobic glycolysis expressed as SUVs ranging from 2.5 to 12. Therefore, for future interventional studies, ^18^F-FDG-PET for therapy response quantification is mandatory before and at the end of any anti-cancer treatment. To verify both BLI and ^18^F-FDG-PET results, all rats underwent also necropsy. During necropsy, tumor tissue was observed in particular on the visceral and parietal peritoneum that was similar to the received information from BLI and ^18^F-FDG-PET imaging studies.

To our knowledge, there are no data comparing ^18^F-FDG-PET and BLI for PM detection of orthotopic colorectal cancer in the rat. However, we observed ^18^F-FDG-PET to be more sensitive than BLI for the detection of early PM in our rat model. Although few data comparing ^18^F-FDG-PET and BLI have yet been obtained in a single animal model, similar findings for BLI and ^18^F-FDG-PET to detect PM of gastric cancer origin in a mouse model was already observed earlier [18].

Based on our findings, BLI and ^18^F-FDG-PET should be used as complementary approaches. BLI is a simple and cost-effective method to monitor the induction and growth of PM. Furthermore, BLI is appropriate for the selection of animals with a similar degree of PM for grouping of as homogeneous treatment groups as possible for interventional studies. For accurate quantification of any therapy response, additional ^18^F-FDG-PET imaging is required before and at the end of any anti-tumor therapy. Although ^18^F-FDG-PET is costly and false-positive results due to focal uptake in non-malignant tissue such as brown fat might be regarded as potential limitations. The high activity in the bladder can also compromise detection and quantitation of tracer accumulation into metastases at a short distance. Nevertheless, ^18^F-FDG-PET is still the most common imaging modality used in oncology for both clinical purposes and experimental setups.

Finally, we have to state that extra-corporeal aerosol generation technology also has disadvantages. The total amount of intraperitoneally deposited aerosol must be determined in advance by means of scintigraphic studies whenever the experimental setup or individual treatment parameters are changed.

Although drugs such as cisplatin, doxorubicin and others can be nebulized with ultrasonic nebulizer systems without degradation it should be borne in mind for future studies, that highly viscous fluids cannot be adequately nebulized with this technology. In addition, the temperature of the fluids in the tank of ultrasonic nebulizers can rise with prolonged use. This can cause heat-sensitive materials to degrade. However, potentially heat-sensitive materials such as proteins and liposomes have already been successfully administered with such devices [21, 22].

A general disadvantage of using a rat model to study pressurized intraperitoneal aerosol therapy is that pressure effects higher than 10 mmHg capnoperitoneum cannot be studied. This is because non-ventilated rats suffer from cardio-respiratory decompensation at capnoperitoneum above 10 mmHg [23].

## Conclusions

The USN with its minimally invasive surgical setup delivers a stable aerosol, is compliant to animal welfare, cost-effective, and therefore suitable for robust and high-throughput species-specific preclinical cancer research with the PM colon cancer model.

## Data Availability

All data and materials as well as furthermore information concerning these are available through contacting the corresponding author.

## Abbreviations

BLI: Bioluminescence imaging
CT: Computed tomography
^18^F-FDG-PET: 2-deoxy-2-[fluorine-18]fluoro-D-glucose PET
IP: Intraperitoneal
LDS: Laser diffraction spectrometry
MIP: Maximum intensity projection
USN: Ultrasonic nebulizer
PET: Positron emission tomography
PM: Peritoneal metastases
PIPAC: Pressurized intraperitoneal aerosol chemotherapy (technology for human use)
SPECT: Single photon emission computed tomography
^99m^Tc: ^99m^technetium sodium pertechnetate
VOI: Volume of interest
